# Periodontal changes after haematopoietic cell transplantation and the role of conditioning regimen intensity

**DOI:** 10.1007/s00520-025-09654-9

**Published:** 2025-06-23

**Authors:** Lucky L. A. van Gennip, Marjolein S. Bulthuis, Gerjon Hannink, Ewald M. Bronkhorst, Stephanie J. M. van Leeuwen, Nicole M. A. Blijlevens, Marie-Charlotte D. N. J. M. Huysmans, Renske Z. Thomas

**Affiliations:** 1 Department of Dentistry, Radboud University Medical Center, Nijmegen, The Netherlands; 2 Department of Medical Imaging, Radboud University Medical Center, Nijmegen, The Netherlands; 3 Department of Hematology, Radboud University Medical Center, Nijmegen, The Netherlands

**Keywords:** Stem cell transplantation, Conditioning regimen intensity, Dentition, Periodontium, Periodontal disease, Longitudinal clinical study

## Abstract

**Purpose:**

To evaluate periodontal health after allogeneic haematopoietic cell transplantation (HCT), and its association with conditioning regimen intensity.

**Methods:**

This single-centre retrospective cohort study included 82 allogeneic HCT recipients between 01/08/2017 and 31/03/2022. Probing pocket depth (PPD), bleeding on probing (BOP), periodontal epithelial surface area (PESA) and periodontal inflamed surface area (PISA) were assessed pre- and post-HCT. Change scores were calculated, and regression models were applied to analyse associations with conditioning intensity. Conditioning regimens were categorised based on intensity as non-myeloablative (NMA), reduced intensity (RIC) or myeloablative (MA).

**Results:**

HCT recipients had a median age of 59 years (IQR 48–66); 63% were male. Median time to HCT was 53 days (IQR 29–89), median follow-up was 279 days (IQR 183–349). Severe periodontitis (≥ 1 site with PPD ≥ 6 mm) was observed in 37% of patients pre-HCT and 20% of patients post-HCT. PPD, BOP, PESA and PISA decreased from pre- to post-HCT, by 0.26 mm [95%CI 0.16;0.37], 8% [95%CI 5;12], 140 mm^2^ [95%CI 89;190] and 123 mm^2^ [95%CI 83;185], respectively. Prevalence of severe periodontitis decreased from pre- to post-HCT in all groups: NMA 50% to 27%, RIC 32% to 19%, MA 31% to 13%. Conditioning intensity was statistically significantly associated with post-HCT PPD and PESA; however, differences were small. No statistically significant differences were observed in post-HCT PISA between conditioning regimens.

**Conclusion:**

Periodontal health improved marginally in the short-term following HCT and supportive oral care. Differences in post-HCT periodontal health between patients conditioned with NMA, RIC, and MA were not clinically relevant.

**Supplementary Information:**

The online version contains supplementary material available at 10.1007/s00520-025-09654-9.

## Introduction

Oral complications, affecting various tissue types, are common in patients undergoing haematopoietic cell transplantation (HCT) [1]. Periodontal tissues, defined as the supporting and investing structures of the teeth, including the root cementum, periodontal ligament, alveolar bone and gingiva, may also be affected [2]. Gingivitis is the mildest form of periodontal disease, is reversible, and does not affect underlying supporting structures of the teeth [3]. Before treatment is initiated, patients with leukaemia may present with gingival bleeding or gingival hyperplasia as a result of disease infiltration [4]. Periodontitis leads to loss of connective tissue and bone support, and is a major cause of tooth loss in adults [3]. The initiation and progression of periodontal disease is determined by a complex interplay between pathogenic microorganisms in the biofilm and the host inflammatory response [5]. Genetic factors and environmental factors, as tobacco use, play a central role [3]. Periodontal disease can occur when the individual’s host response is impaired [3]. In HCT patients, the conditioning regimen induces immunosuppression, which increases the risk of periodontal disease [4]. Graft-versus-Host Disease (GvHD), a common complication after allogeneic HCT, is generally treated with corticosteroid therapy which further induces immunosuppression [6–8]. Another risk for the periodontium includes the oral pain often experienced by HCT recipients, and therefore, maintaining good oral hygiene to remove pathogenic microorganisms can be challenging [9]. On the other hand, increased attention to oral health and the use of antibiotics may decrease the number of periodontal pathogens in the mouth [10]. Professional periodontal care pre-HCT, as recommended by the international guideline for dental care providers, includes dental cleaning, oral hygiene instructions and extraction of severely periodontally involved teeth [11].

Periodontal probing is an important tool to clinically diagnose periodontal health and disease [12]. The clinician places the probe into the periodontal pocket and applies a force to move it apically into the tissue along the tooth surface until displacement of the probe ceases [13]. Studies investigating periodontal health before and after allogeneic HCT are scarce and relatively small [14]. Despite the concern for a negative influence on the periodontium, previous research in allogeneic HCT recipients showed that gingival health and clinical attachment level three months post-HCT were comparable to or even better than pre-HCT [15–17]. In all studies, potential sources of oral infections were treated, and patients received oral hygiene instructions before transplantation. The conditioning regimen is a crucial part in the HCT process, however, the effect of conditioning intensity on periodontal health remains unclear. The aim of this paper was to evaluate periodontal health after transplantation among adult HCT recipients treated at Radboudumc (Nijmegen, the Netherlands), and investigate its association with conditioning intensity.

## Materials and methods

The ethical committee of the Radboudumc confirmed that their approval was not required for this study (2022–13654). The protocol was registered at ClinicalTrials.gov (NCT05595070). STROBE guidelines were followed in the reporting [18].

### Oral care before and after HCT

As part of an oral care program at Radboudumc (Nijmegen, the Netherlands), allogeneic HCT candidates undergo a pre-HCT dental check-up, performed chairside in the dental clinic of the department of Dentistry to identify potential sources of infection. The pre-HCT dental check-up includes a full-mouth periodontal examination [19], unless it is decided to omit periodontal probing due to leukopenia, thrombocytopenia, time constraints or other reasons. After the dental check-up, dental care is provided if deemed necessary. This usually involves extraction of teeth with acute infections (recent pain, sinus tracts, abscesses), endodontic treatment in case of necrotic pulps generally with apical periodontitis, restoration of large cavitated caries lesions, supra- and/or subgingival cleaning and oral hygiene instructions. Supra- and subgingival cleaning are usually performed in the same session as the pre-HCT check-up and do not involve local anaesthesia. Patients are seen for follow-up in the dental clinic of the department of Dentistry approximately five months after HCT, or earlier if there are any oral problems, to monitor their oral status. Dental check-ups after HCT usually include a full-mouth periodontal examination at least once during follow-up. In general, twelve to eighteen months post-HCT, patients are referred back to their own dentist.

### Study population

Patients who received a pre-HCT dental check-up in preparation for allogeneic HCT between August 2017 and March 2022 in Radboudumc were considered for inclusion. As full-mouth periodontal examinations were only routinely performed starting from August 2017, the inclusion period deviates from the registered protocol. Patients included in the study met the following eligibility criteria: 1) at least eighteen years old at transplantation; 2) pre-HCT dental check-up including full-mouth periodontal examination; and 3) at least one follow-up after HCT including full-mouth periodontal examination. Only patients that consented in sharing medical data for research at their first admission to the department of Haematology were included. The first HCT was included for patients who were transplanted twice and periodontally examined before and after both transplantations during the inclusion period. For patients who were periodontally examined twice either before and/or after transplantation, the last periodontal examination before HCT and the first periodontal examination after HCT were included. Patients who were transplanted twice between periodontal examinations were excluded.

### Conditioning regimen

The department of Haematology follows standardised guidelines to determine the optimal conditioning regimen for each patient based on disease- and patient-related factors. Conditioning regimens were categorised into three distinct intensity levels: myeloablative (MA), reduced intensity (RIC) or non-myeloablative (NMA) [20]. Antibacterial prophylaxis was always applied before allogeneic HCT, except when the conditioning regimen involved fludarabine and TBI.

### Periodontal parameters

Study outcomes were pre- to post-HCT change in probing pocket depth (PPD), periodontal epithelial surface area (PESA) and periodontal inflamed surface area (PISA), and its associations with conditioning intensity. PPD and bleeding on probing (BOP) were recorded at six sites per tooth (mesiobuccal, midbuccal, distobuccal, mesiolingual, midlingual, and distolingual) by one trained dentist and two final year dental hygiene students (internships) with a manual periodontal probe (Hu-Friedy Williams). Third molars and dental implants were excluded. PPD was recorded in millimetres and measured from the gingival margin to the bottom of the sulcus. Clinical attachment level could not be calculated due to the absence of gingival margin level assessments at interproximal sites. BOP was scored after probing as yes or no, and used to calculate PISA. PPD and BOP measurements were missing in nine patients on in total 34 pocket-sites (0.1% of total pocket-sites). Mesial and distal surfaces have greater PPD values than corresponding flat surfaces [21]. Therefore, missing values on PPD were replaced by the PPD at the same location (mesial, mid, distal) at the other side of the tooth (buccal, lingual). Missing values on BOP were imputed by mean imputation on tooth level. PESA and PISA were calculated according to the method described by Nesse et al. [22] These quantify root surface area in square millimetres affected by attachment loss and inflamed periodontal tissue, respectively. Severe periodontitis was defined as at least one tooth with a PPD equal or more than 6 mm [23].

### Confounders

The Hematopoietic Cell Transplantation-Comorbidity Index (HCT-CI) was used to evaluate the presence and severity of comorbidities [24]. The follow-up time was calculated as the number of days between periodontal examinations. Smoking behaviour was classified into three categories based on doctors’ notes in electronic health records: never smoked or quit smoking > 10 years before HCT; quit smoking 1–10 years before HCT; current smoker or quit smoking < 1 year before HCT [25].

### Data analysis

Baseline characteristics were retrieved from the electronic health records of Radboudumc. Numbers and proportions were reported for dichotomous and categorical variables, and medians and interquartile ranges for continuous variables.

Periodontal data for teeth extracted after the pre-HCT periodontal examination but before HCT were excluded. Change scores from pre-HCT (after extractions) to post-HCT for PPD, PESA and PISA were presented for descriptive purposes. To determine the confidence interval (CI) around the change score for PPD, a linear mixed effects model was applied with the change score as dependent variable and a random intercept for individuals as independent variable. This comes down to adjusting the standard CI from a paired t-test, for the presence of multiple pairs of measurements in single patients. CIs around change scores for PESA and PISA were calculated using paired t-tests. To reduce dependence on the distributional assumption in determining the CI for the PISA change score, bootstrapping with thousand bootstrap replicates was employed.

In the analyses to assess the association with conditioning intensity, post-HCT scores served as dependent variable and pre-HCT scores (after extractions) as independent variable to enhance precision. To assess the association of conditioning intensity with post-HCT PPD, the six probing sites per tooth were first averaged which yielded a mean PPD per tooth. Linear mixed effects models with random intercepts were applied because of the presence of multiple measurements in single patients. Linear regression models were applied to assess the association of conditioning intensity with post-HCT PESA and PISA. Pre-HCT PPD, PESA and PISA, and conditioning intensity were incorporated in the crude models. Analyses were then adjusted for age at HCT, sex, comorbidities, follow-up time and smoking behaviour. Models involving PESA and PISA as outcome variables were additionally adjusted for number of teeth. The normality of the residuals was determined using residual plots. When residuals were skewed, bootstrapping was applied with thousand bootstrap replicates for more accurate CIs. Regression coefficients with 95% CI were reported and presented in dot-and-whisker plots. Statistical analyses were performed using R (version 4.1.1; R Foundation for Statistical Computing, Vienna, Austria).

## Results

### Study population

**Fig. 1:**
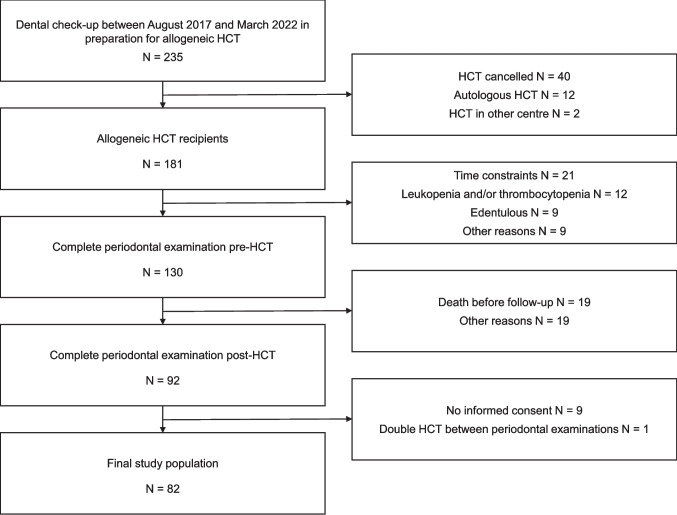
Flowchart of the study population. Reasons for exclusion are shown at the right side of the chart

In total, 235 patients received a dental check-up in preparation for their allogeneic HCT. Of these, 181 actually received an allogeneic HCT in Radboudumc. A full-mouth periodontal examination was conducted in 130 patients, of whom 92 patients were also periodontally examined post-HCT. The final study population consisted of 82 allogeneic HCT recipients (Fig. 1).

### Baseline characteristics

Characteristics are reported in Table 1. Out of 82 HCT recipients, 22 were prepared with NMA regimens, 37 with RIC regimens and 23 with MA regimens. The median age was 59 years (IQR 48–66); 30 (37%) were women. HCT recipients prepared with MA conditioning were younger and had less comorbidities than HCT recipients prepared with RIC and NMA conditioning. Ciprofloxacin was routinely administered as antibacterial prophylaxis to patients conditioned with RIC and MA regimens in the week before transplantation. Patients prepared with NMA regimens (27%) did not routinely receive antibacterial prophylaxis. Following pre-HCT periodontal examination, 24 teeth were extracted in 13 patients (16%). The majority of these patients required one extraction, while one patient required seven extractions. Following pre-HCT periodontal examination, 50% received sub- and supragingival cleaning (ultrasonic and polishing), 33% only supragingival cleaning, and 13% did not receive any professional cleaning. More than half of the patients conditioned with NMA and RIC regimens received sub- and supragingival cleaning, whereas the majority of those conditioned with MA regimens received only supragingival cleaning. Extensive characteristics and GvHD incidence are reported in the supplemental material (Table S1 and Table S2).

**Table 1:** Characteristics of final study population (*n* = 82)

	Total (*n* = 82)	NMA (*n* = 22)	RIC (*n* = 37)	MA (*n* = 23)
Age at HCT, years	59 [48–66]	65 [61–68]	60 [52–66]	46 [36–51]
Sex				
- Female	30 (37%)	12 (55%)	16 (43%)	2 (9%)
- Male	52 (63%)	10 (45%)	21 (57%)	21 (91%)
Medical diagnosis				
- Acute Myeloid Leukaemia	33 (40%)	14 (64%)	8 (22%)	11 (48%)
- Myelodysplastic Syndrome	12 (15%)	8 (36%)	3 (8%)	1 (4%)
- Myeloproliferative Neoplasms	11 (13%)		11 (30%)	
- Lymphoma	8 (10%)		5 (14%)	3 (13%)
- Acute Lymphoblastic Leukaemia	4 (5%)			4 (17%)
- Aplastic Anaemia	4 (5%)		4 (11%)	
- Chronic Myelomonocytic Leukaemia	3 (4%)		3 (8%)	
- Chronic Myeloid Leukaemia	3 (4%)		1 (3%)	2 (9%)
- Other	4 (5%)		2 (5%)	2 (9%)
TBI	33 (40%)	22 (100%)	3 (8%)	8 (35%)
- Dose, Gray	2 [2-8]	2 [2-2]	8 [6-8]	12 [12-12]
Hematopoietic Cell Transplantation-Comorbidity Index (HCT-CI) idem	2 [0–3]	3 [0–3]	2 [1-3]	1 [0–2]
Smoking				
- Never/quit smoking > 10yrs before HCT	54 (66%)	15 (68%)	27 (73%)	12 (52%)
- Former smoker	11 (13%)	5 (23%)	3 (8%)	3 (13%)
- Current/quit smoking < 1 yr before HCT	17 (21%)	2 (9%)	7 (19%)	8 (35%)
Extractions pre-HCT^a^	13 (16%)	3 (14%)	6 (16%)	4 (17%)
- 1	8 (10%)	2 (9%)	3 (8%)	3 (13%)
- 2	3 (4%)	1 (5%)	1 (3%)	1 (4%)
- 3	1 (1%)		1 (3%)	
- 7	1 (1%)		1 (3%)	
Number of teeth at time of HCT idem	26 [22-28]	23 [19-26]	26 [22-28]	27 [26-28]
Brushing method				
- Manual toothbrush	31 (38%)	5 (23%)	14 (38%)	12 (52%)
- Powered toothbrush	41 (50%)	10 (45%)	21 (57%)	10 (43%)
- Combination	8 (10%)	5 (23%)	2 (5%)	1 (4%)
- Unknown	2 (2%)	2 (9%)		
Brushing frequency				
- > 2/day	9 (11%)	4 (18%)	2 (5%)	3 (13%)
- 2/day	50 (61%)	11 (50%)	26 (70%)	13 (57%)
- 1/day	17 (21%)	5 (23%)	6 (16%)	6 (26%)
- Unknown	6 (7%)	2 (9%)	3 (8%)	1 (4%)
Interdental cleaning				
- Yes	52 (63%)	16 (73%)	26 70%)	10 (43%)
- No	23 (28%)	4 (18%)	9 (24%)	10 (43%)
- Unknown	7 (9%)	2 (9%)	2 (5%)	3 (13%)
Periodontal care pre-HCT				
- Sub- and supragingival cleaning	41 (50%)	12 (55%)	21 (57%)	8 (35%)
- Supragingival cleaning	27 (33%)	5 (23%)	10 (27%)	12 (52%)
- No professional cleaning	11 (13%)	4 (18%)	4 (11%)	3 (13%)
- Unknown	3 (4%)	1 (5%)	2 (5%)	
Time between pre-HCT dental check-up and HCT, days	53 [29–89]	55 [35–73]	43 [29–84]	51 [32–95]
Time between HCT and follow-up, days	279 [183–349]	305 [201–393]	272 [190–329]	287 [173–364]

Data are presented as n (%) or median [interquartile range]. Abbreviations: NMA = non-myeloablative conditioning; RIC = reduced intensity conditioning; MA = myeloablative conditioning; HCT = haematopoietic cell transplantation; TBI = total body irradiation. ^a^Reasons for extractions: 5 teeth with acute infections, 5 teeth with severe periodontal disease, 5 teeth with large cavitated caries lesions, 3 retained roots, 1 tooth with necrotic pulp and apical periodontitis, and 5 teeth with necrotic pulps and apical periodontitis combined with severe periodontal disease

### Change of periodontal parameters from pre- to post-HCT

**Fig. 2:**
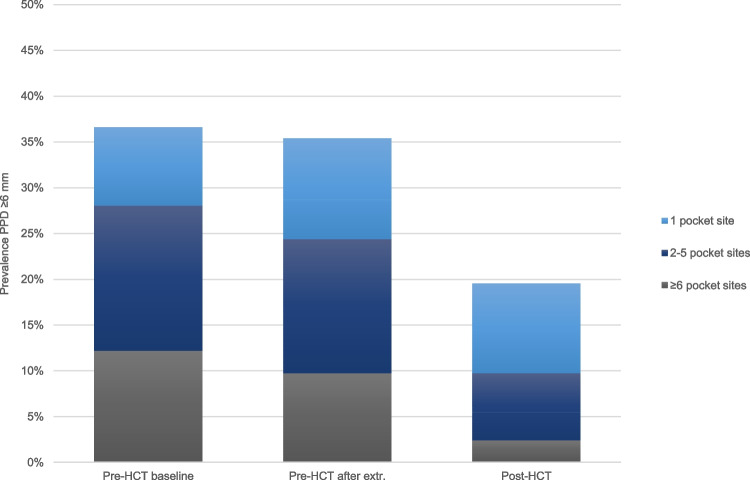
Prevalence of patients with periodontal pocket(s) equal or more than 6 mm according to the amount of pocket sites at three timepoints (pre-HCT, following pre-HCT extractions, post-HCT). Abbreviations: HCT = haematopoietic cell transplantation; PPD = probing pocket depth

Figure 2 displays the prevalence of patients with severe periodontitis at three timepoints. At baseline, 124 teeth in 30 patients (37%) had at least one site with PPD ≥ 6 mm. Following pre-HCT extractions, the prevalence of severe periodontitis decreased to 35% (114 teeth in 29 patients). The prevalence of severe periodontitis further decreased to 20% post-HCT (31 teeth in 16 patients). At baseline, 90% of all pocket sites had a PPD between 0–3 mm, whereas 2% of all pocket sites had a PPD equal or more than 6 mm. These percentages remained the same following pre-HCT extractions. Post-HCT, the percentage of pockets sites with PPD between 0–3 mm increased to 95%, and the percentage of pocket sites with PPD equal or more than 6 mm decreased to 0% (Figure S1).

**Fig. 3:**
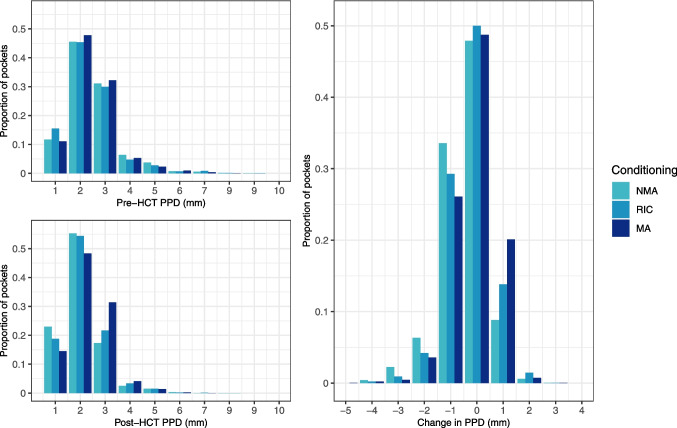
Pre-HCT PPD after extractions, post-HCT PPD and change in PPD (mm) per conditioning intensity. Negative change score implies a reduction in PPD from pre- to post-HCT. Abbreviations: PPD = probing pocket depth; NMA = non-myeloablative conditioning; RIC = reduced intensity conditioning; MA = myeloablative conditioning; HCT = haematopoietic cell transplantation

**Fig. 4:**
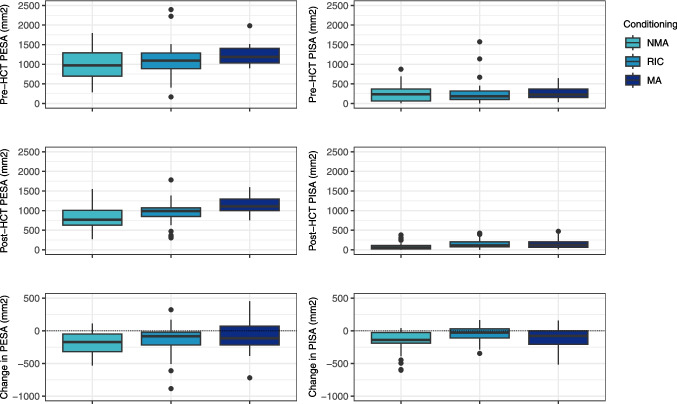
Pre-HCT PESA and PISA after extractions, post-HCT PESA and PISA and change in PESA and PISA (mm2) per conditioning intensity. Negative change score implies a reduction from pre- to post-HCT. The boxes represent the interquartile range, and the upper and lower whiskers extend to the maximum and minimum values excluding outliers. Abbreviations: PESA = periodontal epithelial surface area; PISA = periodontal inflamed surface area; NMA = non-myeloablative conditioning; RIC = reduced intensity conditioning; MA = myeloablative conditioning; HCT = haematopoietic cell transplantation

The mean PPD was 2.44 mm following pre-HCT extractions and decreased to 2.18 mm post-HCT. The mean change score was −0.26 mm [95% CI −0.37;−0.16]. The mean PESA was 1108 mm^2^ following pre-HCT extractions and decreased to 968 mm^2^ post-HCT. The mean change score was −140 mm^2^ [95% CI −190;−89]. The mean PISA was 265 mm^2^ following pre-HCT extractions and decreased to 142 mm^2^ post-HCT. The mean change score was −123 mm^2^ [95% CI −185;−83]. Figure 3 and Fig. 4 illustrate the pre- and post-HCT measurements and change scores for PPD, PESA and PISA categorised by conditioning intensity. The mean percentage BOP was 20% following pre-HCT extractions and decreased to 12% after HCT. The mean change score was −8% [95% CI −12;−5].

### Association of conditioning intensity with post-HCT periodontal health

**Fig. 5:**
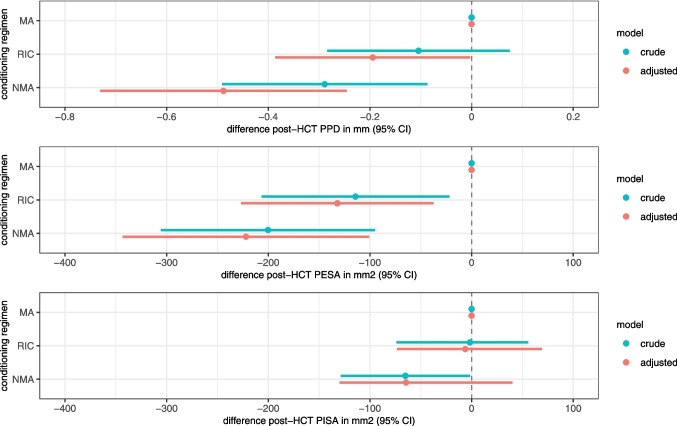
Impact of different conditioning regimen intensities on post-HCT PPD (mm), PESA (mm^2^) and PISA (mm^2^) (corrected for pre-HCT values). MA conditioning serves as the reference category. Whiskers represent 95% confidence intervals. Analyses are adjusted for age, sex, smoking behaviour, comorbidity index, follow-up time, and additionally for number of teeth for PESA and PISA outcomes. Abbreviations: MA = myeloablative conditioning; RIC = reduced intensity conditioning; NMA = non-myeloablative conditioning; PPD = probing pocket depth; PESA = periodontal epithelial surface area; PISA = periodontal inflamed surface area; HCT = haematopoietic cell transplantation; CI = confidence interval

The prevalence of severe periodontitis decreased from pre- to post-HCT across all conditioning groups: from 50 to 27% in NMA patients, from 32 to 19% in RIC patients, and from 31 to 13% in MA patients. Figure 5 displays the impact of conditioning intensity on post-HCT periodontal parameters, adjusted for pre-HCT values and potential confounders. HCT recipients conditioned with RIC regimens had a statistically significant lower mean PPD (−0.19 mm [95% CI −0.38;−0.01]) and statistically significant lower mean PESA (−132 mm^2^ [95% CI −227;−37]) after transplantation compared to HCT recipients conditioned with MA regimens. However, the post-HCT PISA of HCT recipients conditioned with RIC regimens did not differ significantly from the post-HCT PISA of HCT recipients conditioned with MA regimens (−6 mm^2^ [95% CI −73;69]). HCT recipients conditioned with NMA regimens had a statistically significant lower mean PPD (−0.49 mm [95% CI −0.72;−0.26]) and statistically significant lower mean PESA (−222 mm^2^ [95% CI −343;−101]) after transplantation compared to HCT recipients conditioned with MA regimens. The post-HCT PISA of HCT recipients conditioned with NMA regimens did not differ significantly from the post-HCT PISA of HCT recipients conditioned with MA regimens (−65 mm^2^ [95% CI −130;40]).

## Discussion

This retrospective study aimed to evaluate periodontal health after allogeneic HCT, and to study its association with different conditioning regimen intensities. Among 82 allogeneic HCT recipients enrolled in an oral care programme at our institution, PPD, percentage BOP, PESA and PISA decreased marginally from pre- to post-HCT, by 0.26 mm [95% CI 0.16;0.37], 8% [95% CI 5;12], 140 mm^2^ [95% CI 89;190] and 123 mm^2^ [95% CI 83;185], respectively. Associations between conditioning intensity and post-HCT PPD and PESA were statistically significant; however, these differences were not considered clinically relevant. No statistically significant differences in post-HCT PISA were observed between conditioning regimen groups.

A limitation of the study is that PPD and BOP measurements were performed in a clinical setting with no explicit calibration. Inter-examiner variation may exist; however, we postulate that the impact on the overall study results is minimal as all examiners were trained dentists and dental hygiene interns. Also, there was no consistent pattern in which examiners performed pre- or post-HCT measurements. In the present study, all patients received an allogeneic HCT and were part of an oral care program. Due to the lack of a control group, the improvement in periodontal health could result from the HCT procedure and accompanying treatments, the supportive oral health care, or a combination of both. In addition, the proportion of very deep periodontal pockets was low, as severely periodontally involved teeth were regarded as potential source of infection, and the guideline recommendation is to extract them pre-conditioning [11]. Consequently, the interpretation of the results is limited to relatively healthy periodontia. The analyses in the present study were adjusted for all possible known confounders, however, a risk remains that unknown or unmeasured confounders could have biased the results. Socio-economic status may be proposed as a true risk factor for periodontal disease, however, information on socio-economic status was not available [26]. Another limitation of the study is that we could only include patients who were periodontally examined both before and after HCT. Unfortunately, approximately half of the patients who received a dental check-up in preparation for their allogeneic HCT were not subjected to a full-mouth periodontal examination before and/or after HCT for various reasons. Nevertheless, we did not find considerable differences in age, sex, medical diagnosis, conditioning regimen, TBI and comorbidities (Table S3), and thus think that HCT recipients included in this study are representative of the allogeneic HCT population at the Radboudumc in general. This study was retrospective in nature, relying on data collected during routine clinical care. As such, we were dependent on the information that was recorded as part of standard practice. In the clinical setting, it proved impossible to obtain reliable measurements of the gingival margin level at interproximal sites. Therefore, we could not calculate clinical attachment loss. This would be desirable for further research, especially since we have the impression that patients report gingival recessions relatively often after HCT. Superimposition of digital scans with measurements of the gingival location may provide a solution in the future.

A systematic review showed that longitudinal studies on periodontal changes after HCT are both scarce and in general of low methodological quality [14]. In previous studies on periodontal changes after allogeneic HCT, patients received MA conditioning consisting of cyclophosphamide with either busulfan or TBI [16, 17, 20], whereas in the present study different conditioning regimen intensities were applied. Additionally, patients were considerably older in our study. Our findings are in agreement with existing literature, despite differences in conditioning intensities and patients age, indicating that periodontal health does not decline within one year after allogeneic HCT. To explore the potential impact of chemotherapy and/or radiotherapy, we further compared our findings with existing literature in cancer patients in general. In a longitudinal study in patients with solid tumours eligible for adjuvant chemotherapy, mean PPD and percentage BOP decreased after chemotherapy [27]. A study in patients treated with high-dose radiotherapy for head and neck cancer found no change in percentage BOP, while mean PPD decreased significantly [28]. In contrast, the radiotherapy applied to HCT recipients in our study involved TBI, potentially resulting in a lower radiation dose to the periodontium. Nevertheless, our results on PPD align with previous studies.

The attention for oral hygiene at our department may have contributed to improvement of periodontal health. All HCT recipients received oral hygiene instructions, and the vast majority received professional dental cleaning before transplantation. As periodontal health is related to the presence of plaque, improved brushing by HCT recipients will induce improvement of periodontal health, which is reflected in a decrease of PPD, percentage BOP, PESA and PISA after transplantation [29]. On top of that, antibiotics are commonly prescribed by haematologists because of immunosuppression. These antibiotics may reduce pathogenic microorganisms in the biofilm. The incidence of chronic GvHD varied considerably among HCT recipients conditioned with NMA, RIC and MA regimens. As NMA and RIC regimens are less effective in eradicating the recipient’s immune system, they are associated with a higher risk of GvHD, often necessitating prolonged use of immunosuppressive medication [20]. Nevertheless, differences in post-HCT PPD, PESA, and PISA across conditioning intensities were not considered clinically relevant. More than half of the patients conditioned with NMA and RIC regimens received both sub- and supragingival cleaning, whereas the majority of those conditioned with MA regimens received only supragingival cleaning. Periodontal care may have acted as a mediator, as the type of cleaning was influenced by pre-HCT periodontal and likely contributed to differences in post-HCT periodontal health.

## Conclusions

Periodontal health showed marginal improvement over the median nine-month follow-up period following allogeneic HCT and supportive oral care. Differences in post-HCT periodontal health between patients conditioned with NMA, RIC, and MA regimens were not clinically relevant.

## Supplementary Information

Below is the link to the electronic supplementary material. 

ESM1 (DOCX 32.5 KB)

## Data Availability

The data that support the findings of this study are available from the corresponding author upon reasonable request.
