# Supplementary material for: Periodontal changes after haematopoietic cell transplantation and the role of conditioning regimen intensity
Source: Support Care Cancer. 2025 Jun 23;33(7):612. doi: 10.1007/s00520-025-09654-9 (PMC12185657; doi:10.1007/s00520-025-09654-9)
Supplement: Supplementary file 1 — (DOCX 32.5 KB) [file 520_2025_9654_MOESM1_ESM.docx]

**Periodontal changes after haematopoietic cell transplantation and the role of conditioning regimen intensity**

Lucky L.A. van Gennip^a^; Marjolein S. Bulthuis^a^; Gerjon Hannink^b^; Ewald M. Bronkhorst^a^; Stephanie J.M. van Leeuwen^a^; Nicole M.A. Blijlevens^c^; Marie-Charlotte D.N.J.M. Huysmans^a^; Renske Z. Thomas^a^

^a^ Department of Dentistry, Radboud university medical center, Nijmegen, The Netherlands.

^b^ Department of Medical Imaging, Radboud university medical center, Nijmegen, The Netherlands.

^c^ Department of Hematology, Radboud university medical center, Nijmegen, The Netherlands.

Corresponding author:

Lucky L.A. van Gennip

Department of Dentistry, Radboudumc

Philips van Leydenlaan 25, 6525 EX Nijmegen

The Netherlands

Email address: Lucky.vanGennip@radboudumc.nl

ORCID: 0000-0003-0446-9060

# Supplemental material

## Table S1: Extensive baseline and HCT characteristics of final study population (n=82).

|  | Total  (n=82) | NMA  (n=22) | RIC  (n=37) | MA  (n=23) |
| --- | --- | --- | --- | --- |
| First allogeneic HCT | 81 (99%) | 22 (100%) | 36 (97%) | 23 (100%) |
| Donor type   - Sibling - MUD - Haplo - MMUD - Cord blood | 16 (20%)  53 (65%)  1 (1%)  11 (13%)  1 (1%) | 5 (23%)  13 (59%)  4 (18%) | 8 (22%)  24 (65%)  4 (11%)  1 (3%) | 3 (13%)  16 (70%)  1 (4%)  3 (13%) |
| Donor sex   - Female - Male - Male / Male (Cord blood) | 24 (29%)  57 (70%)  1 (1%) | 8 (36%)  14 (64%) | 11 (30%)  25 (68%)  1 (3%) | 5 (22%)  18 (78%) |
| Stem cell source   - Blood - Bone Marrow - Cord blood | 75 (91%)  6 (7%)  1 (1%) | 22 (100%) | 32 (86%)  4 (11%)  1 (3%) | 21 (91%)  2 (9%) |
| T cell depletion   - No depletion - CD34 - ptCy | 54 (69%)  1 (1%)  27 (33%) | 22 (100%) | 32 (87%)  5 (14%) | 1 (4%)  22 (96%) |
| Disease Risk Index   - Very high - High - Intermediate - Unknown | 4 (5%)  38 (46%)  36 (44%)  4 (5%) | 11 (50%)  11 (50%) | 3 (8%)  15 (41%)  15 (41%)  4 (11%) | 1 (4%)  12 (52%)  10 (43%) |
| BMI, kg/m^2^ | 25 [23-28] | 25 [24-26] | 26 [23-28] | 25 [24-28] |
| Interdental cleaning method   - Wooden sticks, n - Softpicks, n - Brushes, n - Floss, n - Other method, n | 15  8  17  20  3 | 5  1  7  4  1 | 9  5  7  10  2 | 1  2  3  6 |
| Interdental cleaning frequency   - Daily, n - <1/day, n - Unknown, n | 30  11  11 | 11  3  2 | 13  5  8 | 6  3  1 |
| Visit oral health care providers   - Regularly - Irregularly / not recently - Unknown | 72 (88%)  7 (9%)  3 (4%) | 20 (91%)  1 (5%)  1 (5%) | 34 (92%)  2 (5%)  1 (3%) | 18 (78%)  4 (17%)  1 (4%) |

Data are presented as n (%) or median [interquartile range].
Abbreviations: NMA = non-myeloablative conditioning; RIC = reduced intensity conditioning; MA = myeloablative conditioning; HCT = haematopoietic cell transplantation; MUD = matched unrelated donor; Haplo = haploidentical donor; MMUD = mismatched unrelated donor; BMI = body mass index.

## Table S2: Graft-versus-Host Disease characteristics of final study population (n=82).

|  | Total  (n=82) | NMA  (n=22) | RIC  (n=37) | MA  (n=23) |
| --- | --- | --- | --- | --- |
| aGvHD Harris   - Grade I - Grade II - Grade III - Grade IV | 47 (57%)  18  22  6  1 | 13 (59%)  2  10  1 | 23 (62%)  11  8  4 | 11 (48%)  5  4  1  1 |
| - Classical aGvHD - Late onset aGvHD | 35  12 | 10  3 | 15  8 | 10  1 |
| aGvHD therapy   - Prednisolone 0.5 mg/kg - Prednisolone 1 mg/kg - Prednisolone 2 mg/kg - Triamcinolone | 12  19  12  4 | 2  8  3 | 7  7  7  2 | 3  4  2  2 |
| cGvHD   - Classic - Overlap Syndrome - Limited - Extensive - NIH = 0 - Mild - Moderate - Severe | 22 (27%)  21  1  11  11  2  3  10  7 | 10 (45%)  10  4  6  1  2  3  4 | 11 (30%)  11  6  5  1  1  6  3 | 1 (4%)  1  1  1 |

Data are presented as n (%).
Abbreviations: NMA = non-myeloablative conditioning; RIC = reduced intensity conditioning; MA = myeloablative conditioning; aGvHD = acute Graft-versus-Host Disease; cGvHD = chronic Graft-versus-Host Disease; NIH = National Institutes of Health.

## Table S3: Baseline and HCT characteristics of eligible patients who received dental check-up in preparation for their allogeneic HCT and consented in sharing medical data (n=163) and patients included in the final study population (n=82).

|  | Patients who received dental check-up in preparation for their allogeneic HCT and consented in sharing medical data  (n=163) | Final  study population  (n=82) |
| --- | --- | --- |
| Age at HCT, years | 58 [46-64] | 59 [48-66] |
| Sex   - Female - Male | 63 (39%)  100 (61%) | 30 (37%)  52 (63%) |
| Medical diagnosis   - Acute Myeloid Leukaemia - Myelodysplastic Syndrome - Myeloproliferative Neoplasms - Lymphoma - Acute Lymphoblastic Leukaemia - Aplastic Anaemia - Chronic Myelomonocytic Leukaemia - Chronic Myeloid Leukaemia - Other | 55 (34%)  25 (15%)  20 (12%)  18 (11%)  14 (9%)  13 (8%)  6 (4%)  4 (3%)  8 (5%) | 33 (40%)  12 (15%)  11 (13%)  8 (10%)  4 (5%)  4 (5%)  3 (4%)  3 (4%)  4 (5%) |
| Conditioning regimen   - NMA - RIC - MA | 47 (29%)  67 (41%)  49 (30%) | 22 (27%)  37 (45%)  23 (28%) |
| TBI   - Dose, Gray | 78 (48%)  0 [0-2] | 33 (40%)  0 [0-2] |
| Hematopoietic Cell Transplantation-Comorbidity Index (HCT-CI) | 1 [0-3] | 2 [0-3] |

Data are presented as n (%) or median [interquartile range].
Abbreviations: HCT = haematopoietic cell transplantation; NMA = non-myeloablative conditioning; RIC = reduced intensity conditioning; MA = myeloablative conditioning; TBI = total body irradiation.

## Figure S1: Percentage of pocket sites with PPD 0-3 mm, PPD 4-5 mm and PPD ≥6 mm at three timepoints (pre-HCT, following pre-HCT extractions, post-HCT). Abbreviations: HCT = haematopoietic cell transplantation; PPD = probing pocket depth.
